# DNA Methylation Can Mediate Local Adaptation and Response to Climate Change in the Clonal Plant *Fragaria vesca*: Evidence From a European-Scale Reciprocal Transplant Experiment

**DOI:** 10.3389/fpls.2022.827166

**Published:** 2022-02-28

**Authors:** Iris Sammarco, Zuzana Münzbergová, Vít Latzel

**Affiliations:** ^1^Institute of Botany, Czech Academy of Sciences, Průhonice, Czechia; ^2^Department of Botany, Faculty of Science, Charles University, Prague, Czechia

**Keywords:** adaptation, survival, 5-azacytidine, climate change, latitudinal gradient, clonal plant, epigenetics

## Abstract

The ongoing climate crisis represents a growing threat for plants and other organisms. However, how and if plants will be able to adapt to future environmental conditions is still debated. One of the most powerful mechanisms allowing plants to tackle the changing climate is phenotypic plasticity, which can be regulated by epigenetic mechanisms. Environmentally induced epigenetic variation mediating phenotypic plasticity might be heritable across (a)sexual generations, thus potentially enabling rapid adaptation to climate change. Here, we assessed whether epigenetic mechanisms, DNA methylation in particular, enable for local adaptation and response to increased and/or decreased temperature of natural populations of a clonal plant, *Fragaria vesca* (wild strawberry). We collected ramets from three populations along a temperature gradient in each of three countries covering the southern (Italy), central (Czechia), and northern (Norway) edges of the native European range of *F. vesca*. After clonal propagation and alteration of DNA methylation status of half of the plants *via* 5-azacytidine, we reciprocally transplanted clones to their home locality and to the other two climatically distinct localities within the country of their origin. At the end of the growing season, we recorded survival and aboveground biomass as fitness estimates. We found evidence for local adaptation in intermediate and cold populations in Italy and maladaptation of plants of the warmest populations in all countries. Plants treated with 5-azacytidine showed either better or worse performance in their local conditions than untreated plants. Application of 5-azacytidine also affected plant response to changed climatic conditions when transplanted to the colder or warmer locality than was their origin, and the response was, however, country-specific. We conclude that the increasing temperature will probably be the limiting factor determining *F. vesca* survival and distribution. DNA methylation may contribute to local adaptation and response to climatic change in natural ecosystems; however, its role may depend on the specific environmental conditions. Since adaptation mediated by epigenetic variation may occur faster than *via* natural selection on genetic variants, epigenetic adaptation might to some degree help plants in keeping up with the ongoing environmental crisis.

## Introduction

Understanding the basis of plants ability to cope with rapidly changing environment is crucial in predicting and mitigating the consequences of the ongoing climate crisis. Plants face rapid climatic and environmental change with three commonly accepted mechanisms: by moving to more favorable conditions, by genetically adapting to the changed environment, or by adjusting their phenotypes to the changed environment. Considering that the sessile lifestyle limits plant movement to novel environments, the “escape” strategy can have only limited effect (Loarie et al., 2009). Furthermore, natural selection may not be quick enough to enable plant populations to adapt to changing environment (Visser, 2008; Hoffmann and Sgrò, 2011; Merilä, 2012; Chevin et al., 2013; Carlson et al., 2014; Botero et al., 2015). Phenotypic plasticity can be thus on the forefront as the most powerful mechanism of plants to tackle the changing climate (Nicotra et al., 2010; Hoffmann and Sgrò, 2011).

Phenotypic plasticity can be mediated by epigenetic mechanisms, such as DNA methylation, which affect phenotypes by regulating gene activity and that may result in locally adapted phenotypes (Merilä and Hendry, 2014). Epigenetic variation can be triggered by environmental variation, arise stochastically (i.e., epimutations) or can be driven by genetic variants (Ahmed et al., 2011; Zhang et al., 2013, 2018; Johannes and Schmitz, 2019). Genetically induced epigenetic variants are completely under genetic control and arise slowly within a population because of the low rate of genetic changes (Richards, 2006). On the contrary, by acting independently of genetic variants, environmentally and stochastically induced epigenetic variants can quickly create novel phenotypes. Importantly, epigenetic variation can be heritable across multiple generations, thus representing another source of heritable variation on which natural selection can operate (Richards, 2006; Hauser et al., 2011; McNamara et al., 2016; Sobral et al., 2021).

Heritable epigenetic variation triggered either by the environment or originating by stochastic epimutations can play a crucial role in the adaptation of clonal populations (Latzel and Klimešová, 2010; Richards et al., 2012; Verhoeven and Preite, 2014; Dodd and Douhovnikoff, 2016; Latzel et al., 2016; Münzbergová et al., 2019; Shi et al., 2019). In fact, clonal species usually form populations with limited standing genetic variation, which can remarkably slow down their adaptation to the rapidly changing environment (Dodd and Douhovnikoff, 2016). As heritability of epigenetic variants seems to be more prominent across clonal than sexual generations (reviewed in Feng et al., 2010; Anastasiadi et al., 2021), heritable epigenetic variation can compensate the lack of standing genetic variation in clonal populations (Dodd and Douhovnikoff, 2016; González et al., 2016; Rendina González et al., 2018; Zhang et al., 2018).

Recent studies suggested that mechanisms enabling epigenetic adaptation can depend on environmental conditions (Fan et al., 2013; Ci et al., 2015; Li et al., 2016; Hossain et al., 2017; Tang et al., 2018). For example, in many plant species, heat but not cold stress is accompanied by a global reduction of DNA methylation (i.e., hypomethylation; Ci et al., 2015; Li et al., 2016; Hossain et al., 2017), suggesting that the role of DNA methylation in response to different temperatures can vary.

However, despite DNA methylation seems to be a potent mechanism enabling rapid adaptation to climate change, solid evidence of its importance in plant adaptation in natural ecosystems is still missing. In fact, studies have traditionally attempted to provide evidence of the role of DNA methylation in local adaptation only indirectly, by associating patterns of epigenetic variation of natural populations to local conditions (Dubin et al., 2015; Platt et al., 2015). However, this approach can provide ambiguous evidence of the role of DNA methylation in local adaptation, as the observed epigenetic patterns might be linked to genetic variation (i.e., genetic rather than epigenetic adaptation), or might not be heritable and thus evolutionarily relevant. Such limitations can be bypassed by using reciprocal transplant experiments involving experimental alteration of plant epigenomes before transplantation to their home and away environments. Comparing the fitness of plants with altered DNA methylation with those with natural DNA methylation can serve as a test of epigenetically driven local adaptation (e.g., Herden et al., 2019). Reciprocal transplant experiments are indeed traditional tests for the study of local adaptation (e.g., Ågren and Schemske, 2012). When including the experimental alteration of epigenomes of clones of the same plants, reciprocal transplant experiments can provide a direct evidence of the role of epigenetic mechanisms in local adaptation and/or response to changing climate, without the confounding effect of underlying genetic variation. However, these studies are currently very rare, and to our knowledge, there is only one other study employing such an approach in field conditions (Herden et al., 2019).

In our study, we asked whether epigenetic mechanisms, DNA methylation in particular, enable for local adaptation of natural populations of a clonal species, *Fragaria vesca* (wild strawberry). We also tested whether potential DNA methylation driven by local conditions alters plant response to climate change, to increased and/or decreased temperature, respectively. We chose *F. vesca* as it occurs in broadly heterogeneous habitats, it has wide geographic distribution and extensive clonal propagation. To fulfil our aims, we collected plants (ramets) from three sites along a temperature gradient in each of three countries covering the southern (Italy), central (Czechia), and northern (Norway) edges of the native European range of *F. vesca*. We moved the ramets to a common garden where we let them clonally propagate for one season. We used two ramets from each clone and planted them individually in a greenhouse and let them to produce several offspring ramets. In half of these ramets (hereafter plants), we altered the DNA methylation status by using the demethylating agent 5-azacytidine (5-azaC), employing the foliar application method described by Puy et al. (2018). In the following spring 2019, we transplanted plants to their home locality and to the two other localities (away) within the country of their origin. Three months later, we recorded their survival, their leaf number, and size and damage due to herbivory.

We asked three specific questions to address three hypotheses: (1) Is there evidence of local adaptation of *F. vesca* populations? We hypothesize that our populations show evidence of local adaptation, that is, that the local plants will have higher survival and/or biomass, and/or reduced herbivory damage than the non-local plants. (2) Is local adaptation mediated by DNA methylation? We hypothesize that local adaptation is under DNA methylation control, that is, that the local plants with altered DNA methylation will have reduced survival and/or biomass, and/or increased herbivory damage than the local plants with natural DNA methylation. (3) Is adaptation to warm conditions mediated differently than to cold conditions? We hypothesize that DNA methylation plays different roles in adaptation to warm and/or cold conditions (e.g., Pan et al., 2011; Ci et al., 2015; see above), that is, that effects of altered DNA methylation on survival, biomass, and/or herbivory damage will be influenced by climatic conditions and/or regions (countries) of plant origin.

## Materials and Methods

### Study Species

*Fragaria vesca* L., Rosaceae, is an herbaceous perennial species growing in disturbed and degraded forests, forest edges, and meadows. It has a wide geographic distribution: it occurs throughout Europe, northern Asia, North America, and northern Africa (Darrow, 1966). It is able to reproduce both sexually through seeds and clonally by producing stolons although its sexual reproduction is very rare in natural conditions (Schulze et al., 2012).

### Sites Selection

We conducted the study at nine sites across three European countries: Italy, Czechia, and Norway (Table 1). We selected the countries to include populations from the southern (Italy) and northern (Norway) limits of the native range of *F. vesca* distribution and populations from the core of its distribution range (Czechia) in Europe. Within each country, we selected three sites to be distributed along a climatic gradient ranging from warmest to coldest mean annual temperatures (defined as: warm, intermediate, and cold sites), usually from lowland to mountain regions. Therefore, hereafter we use term “temperature of origin” of plants, although we are aware that selected populations differed also in other environmental factors. The sizes of the selected populations ranged from 12 to 800 m^2^ (Table 1).

**Table 1:** Temperature range, mean annual temperature, elevation, population size (m^2^), and location of selected populations in the three European countries.

Country (distribution range)	Temperature range	2011–2018 mean T (°C)	Elevation (m)	Population size (m^2^)	Location
Italy (southern edge)	Warm	10.34	468	45	46.471°N, 11.343°E
	Intermediate	3.55	1,436	156	46.725°N, 11.422°E
	Cold	2.45	1905	42	46.337°N, 11.790°E
Czechia (center)	Warm	10.62	201	140	50.399°N, 14.412°E
	Intermediate	9.49	306	400	50.459°N, 14.785°E
	Cold	5.59	875	800	50.811°N, 15.359°E
Norway (northern edge)	Warm	2.88	597	160	60.895°N, 7.349°E
	Intermediate	1.50	818	12	61.036°N, 9.079°E
	Cold	1.25	323	60	60.821°N, 8.706°E

### Climatic Data

We sourced climatic data from the European gridded dataset E-OBS, available through the C3S Climate Data Store (CDS) website (https://cds.climate.copernicus.eu/cdsapp#!/home; Cornes et al., 2018). We retrieved the mean of daily values of temperature over the years 2011–2018, with a horizontal resolution at 0.1 × 0.1° (v20.0e).

### Plant Collection, Cultivation, and 5-azaC Treatment

We collected 5 to 7 ramets from each of the nine populations (sites) between May and July 2018 and transported them to the common garden of the Institute of Botany of the Czech Academy of Sciences in Průhonice, Czechia (49.994°N, 14.566°E). The collected ramets were wrapped in wet paper cloth, placed in plastic bags, and transported in a refrigerating box at 8°C. Individual ramets were planted 1 to 10 days after collection in 70 × 40 × 20 cm trays filled with a commercial mixture of compost and sand, under a shading coverage (reduction of light for 50% to simulate natural light levels at most of the localities). In October 2018, we collected two comparable offspring ramets (F1 clonal offspring, connected with original maternal ramet *via* stolon) from each of the maternal plants and planted them individually in separate 35 × 22 × 5 cm trays placed in a greenhouse tempered at 20/15°C (day/night), 14 h photoperiod to precultivate plant material for the transplant experiment, see later. We sprayed half of the plants twice a week with an aqueous solution of 5-azaC (50 μM in the first 3 weeks of the treatment, and 100 μM in the following months) and a surfactant (1 ml Silwet Star—AgroBio Opava s.r.o./ 1 l solution), from the beginning of February 2019 to late May 2019. This approach allows to obtain similar demethylation effects as the original method based on the germination of seeds in a 5-azaC solution, however avoiding the unwanted side effects usually observed in the original method (e.g., underdeveloped root systems and high mortality of treated plants; Puy et al., 2018). In order to control for potential unknown effects of the surfactant, we sprayed the other half of the plants only with water and surfactant (1 ml Silwet Star/1 l solution). One month prior to the transplant experiment (April 2019), we moved all plants back to the common garden. For transplantation to the field sites, we preferred using the youngest ramets, that is, ramets developed after the start of the demethylation treatment. Moreover, a global DNA methylation analysis confirmed overall demethylating effect of 5-azaC on treated plants (see later).

We also determined the genetic relatedness of a random subset of transplanted plants using whole-genome SNP data to test whether the effect of 5-azaC differed between genotypes and to determine genetic diversities of the populations (see later; Sammarco et al., unpublished).

### Global DNA Methylation Quantification

We quantified global DNA methylation level of a subset of the 5-azaC-treated plants (*N* = 11) and of the respective ramets with natural DNA methylation (*N* = 11), that is, not treated plants by 5-azaC, with the MethylFlash Methylated DNA Quantification Kit-Colorimetric (Epigentek) following the manufacturer’s instructions. Briefly, we extracted genomic DNA using the Qiagen DNeasy Plant Mini Kit and used between 50 and 200 ng of input DNA per reaction. After binding the DNA to the strip wells, we incubated the reaction with capture and detection antibodies allowing the quantification of global DNA methylation through an ELISA-like reaction at 450 nm.

We then normalized the DNA methylation level of 5-azaC plants ( $5mC{\%}_{5\mathrm{azaC}})$ with that of ramets with natural methylation ( $5mC{\%}_{\mathrm{Ctrl}}$ ), following the “Relative Quantification” method as in the manufacturer’s instructions, and using the following formula:

$$\mathrm{Normalized}\;5mC{\%}_{5\mathrm{azaC}}=\frac{5mC{\%}_{5\mathrm{azaC}}}{5mC{\%}_{\mathrm{Ctrl}}}\times 100\%$$

### Reciprocal Transplant Experiment

Between late May and early June 2019, we collected individual rooting ramets from the precultivated plants. We separated them, wrapped in wet paper cloth, placed in plastic bags, and transported in a refrigerating box at 8°C to the target localities within 1–7 days. We standardized all ramets to consist of two leaves, to avoid different plant sizes at the transplantation time. The transplantation sites included the home locality of the maternal plants and the other two away sites within the country of their origin, for example, plants from the warm localities were transplanted to the warm localities (home sites), intermediate and cold localities (away sites) within each country (Supporting Information, Supplementary Table S1). Accordingly, we planted ramets of local plants and ramets of other two populations from the same country in each site. We distinguish a target site (site where the plants were transplanted) and a site of origin (site where the original ramet was collected). We consider plants transplanted back to their site of origin as growing in their *home* environment (i.e., origin site = target site), whereas plants transplanted to different sites as growing in *away* sites (i.e., origin site ≠ target site). In each target site, we planted between 49 and 116 plants. The number of transplanted plants depended on the availability of plant material at the collection time (the specific number of transplanted ramets is presented in Supplementary Table S1). Across all target sites, we evenly distributed 2 to 4 ramets originated from the same maternal plant. However, in minority of cases we transplanted only 1 ramet for a specific site (according to the availability of plant material). In order to control for the unknown transplantation effect and precultivation of plants in the common garden of the Institute, we also replanted between 8 and 10 local plants of *F. vesca* population found at the transplantation site at each locality except for the cold locality in Czechia, where we could not find any local plants at the time of transplantation. We dug out the local plants, standardized them to consist of two leaves (i.e., similar to the precultivated ramets), and replanted them immediately back to the same locality. Thus, these plants had not been transplanted across different localities and had not been precultivated in the common garden (hereafter referred to as *replanted local plants*). We planted the ramets at least 10 cm apart from each other in a randomized grid. Each ramet was labelled with a unique and anonymized code. We watered all plants immediately after planting, but did not provide any additional treatment later during the growing season. Together, we transplanted 801 ramets across all localities.

### Measurements

In September 2019, that is, 3 months after planting, we recorded survival of transplanted ramets, number of leaves, length of the longest leaf (cm), herbivory damage (5 categories: “0” no damage, “1” 1–5% of leaf area removed, “2” 6–25%, “3” 26–50%, “4” 51–75%, “5” >75%), number of flowers and fruits, and number of stolons and ramets. For each plant, we estimated its biomass as the total number of leaves of transplanted (maternal) ramet multiplied by the length of its longest leaf. Since only few plants flowered, fruited, or produced stolons and offspring ramets (<4%), we did not analyze these data.

### Data Analysis

To test for evidence of local adaptation of *F. vesca* populations and for the role of DNA methylation in local adaptation, we tested the effects of country, temperature of origin, home/away, and 5-azaC treatment on plant survival, biomass, and herbivory damage. To test for the role of DNA methylation in plants from different climatic conditions and/or countries, we replaced both temperature of origin and home/away with the temperature distance between origin and target sites. We calculated temperature distance as the difference between the long-term mean average temperature of the site of origin (over the years 2011–2017) and the short-term mean average temperature of the target site (i.e., after the transplantation time, 2019). We calculated all the temperature averages using only the months included in the growing season (from June to August). We also repeated the same test replacing temperature distance with precipitation distance, and we found similar significant interactions as for the temperature distance (Supplementary Note).

We could not test the effects of both temperature and precipitation distances together since we found these variables to be highly correlated (*p* = 0.033, *R* = −0.41). We then chose to show temperature as climatic variable for the above-mentioned test in the main text since the field sites were selected primarily along a temperature gradient.

We used survival, herbivory damage, and biomass as dependent variables. We log-transformed biomass to fit the assumptions of normality. We coded herbivory as 0 and 1 (respectively, 0–5 and > 5% of area removed) as the data had strongly bimodal distribution. We analyzed survival data using the complete dataset, while we used only the surviving plants when analyzing biomass and herbivory damage.

To analyze the data, we used mixed effect models with maternal plant code as random factor. To account for the effect of plant biomass on herbivory, we included biomass as a covariate in the models testing herbivory damage.

We also tested for possible unknown side effects induced by the transplantation and precultivation of the plants in the common garden, by comparing survival and biomass of plants with natural DNA methylation to those of replanted local plants (Supplementary Note).

We tested the binary variables (survival and herbivory damage) with Generalized linear mixed models (GLMMs; binomial distribution and logit link function), using the LME4 package for R (Bates et al., 2015). We tested the biomass index using Linear mixed models (LMMs) with the LMERTEST package (Kuznetsova et al., 2017). We performed all analyses in RStudio, using R 3.6.2 (R Core Team, 2017).

To test whether the effect of 5-azaC differed between genotypes, we partly modified the approach used in Münzbergová et al. (2019). We calculated the genetic relationship matrix for each individual plant from whole-genome SNP data (Sammarco et al., unpublished) with the SNPRelate package for R (Zheng et al., 2012). For each genotype and separately for each locality, we calculated average proportional change for both survival and biomass (“Trait”) after removal of DNA methylation compared to plants with natural DNA methylation, as:

$$\mathrm{Proportional change}=\frac{{\mathrm{Trait}}_{\mathrm{Ctrl}}-{\mathrm{Trait}}_{5\mathrm{azaC}}}{{\mathrm{Trait}}_{\mathrm{Ctrl}}+{\mathrm{Trait}}_{5\mathrm{azaC}}}$$

We calculated pairwise differences in response to removal of DNA methylation of each genotype in each target locality using Euclidean distance. We tested the correlation between the genetic relationship matrix and matrix of distances in response to removal of DNA methylation using the vegan R package (Oksanen et al., 2020). We also repeated the tests with partial Mantel tests using four different matrices as covariates, accounting for the temperature distance of either the origin or target sites, and for origin or target site (calculated as: “0” when the two genotypes had the same origin or target site, respectively; “1” when their origin or target sites were different). The tests showed no significant results in neither case and are thus only shown in (Supporting Information, Supplementary Table S2).

## Results

### Reduced Global DNA Methylation in 5-azaC-Treated Plants

In order to assess the actual alteration of DNA methylation in plants treated with 5-azaC, we quantified global DNA methylation level of a random subset of the 5-azaC-treated plants (*N* = 11) and of the same genotypes with natural DNA methylation (*N* = 11). The mean methylation level of 5-azaC-treated plants was 26.85% lower (SE ± 8.00) than control plants (*t* = −3.352, *p* < 0.001), after excluding four outliers whose DNA methylation level exceeded 100% methylation change compared to the control plants.

### Is There Evidence of Local Adaptation of *F. vesca* Populations? (Hypothesis 1)

To test for evidence for local adaptation of *F. vesca* populations and for the role of DNA methylation in local adaptation, we tested the effects of country, temperature of origin, home/away, and 5-azaC treatment on plant survival, biomass, and herbivory damage (Table 2).

**Table 2:** Test of local adaptation (Hypothesis 1; A) and role of DNA methylation in local adaptation (Hypothesis 2; B).

	Survival			Biomass			Herbivory damage		
	d.f.	F	*P*	d.f.	F	*P*	d.f.	F	*P*
**A**									
Country (C)	2	**4.30**	**<0.001**	2	**3.81**	**0.024**	2	0.11	0.537
Temperature (T)	2	0.37	0.331	2	1.57	0.210	2	0.69	0.221
Home/Away (H/A)	1	0.00	0.950	1	0.49	0.483	1	0.36	0.145
C × T	4	0.22	0.419	4	1.84	0.125	4	0.66	0.298
C × H/A	2	6.78	0.293	2	1.56	0.212	2	0.22	0.716
T × H/A	2	25.10	0.081	2	**12.48**	**<0.001**	2	4.31	0.848
C × T × H/A	4	**1.04**	**0.004**	4	**3.08**	**0.016**	4	1.70	0.089
**B**									
5-azaC	1	1.04	0.271	1	0.27	0.605	1	**5.81**	**0.005**
C × 5-azaC	2	**1.99**	**<0.001**	2	1.64	0.195	2	**0.02**	**0.004**
T × 5-azaC	2	0.83	0.255	2	1.36	0.258	2	0.33	0.694
H/A × 5-azaC	1	1.78	0.417	1	0.53	0.467	1	2.11	0.278
C × T × 5-azaC	4	0.25	0.557	4	1.48	0.207	4	0.99	0.139
C × H/A × 5-azaC	2	0.36	0.160	2	2.90	0.056	2	0.13	0.580
T × H/A × 5-azaC	2	2.44	0.064	2	**6.44**	**0.002**	2	1.31	0.086
C × T × H/A × 5-azaC	4	0.08	0.433	2	2.11	0.123	2	0.90	0.399

Effects of country (Country, C), temperature of origin (Temperature, T), home/away (Home/Away, H/A), 5-azaC treatment (5-azaC), and their interactions on plant survival, biomass, and herbivory damage. *N* = 730 (survival), *N* = 403 (biomass, herbivory damage). D.f.: degrees of freedom. Significant values (*p* ≤ 0.05) are shown in bold. Estimates of the effects can be found in (Supporting Information, Supplementary Table S3).

**Figure 1:**
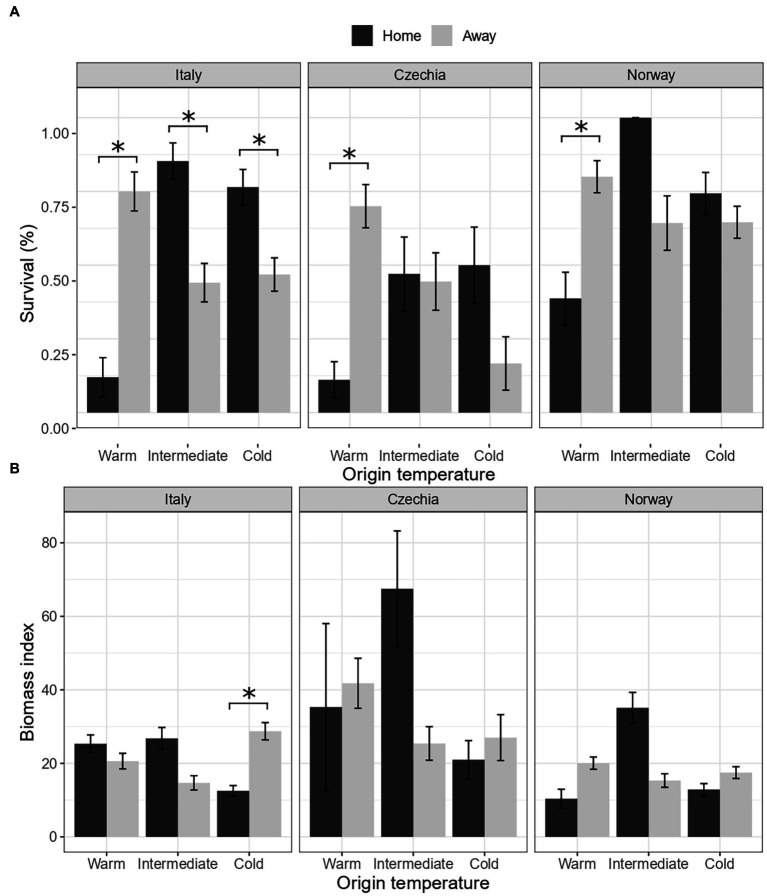
Is there evidence of local adaptation of *F. vesca* populations (Hypothesis 1)? Effects of country, origin temperature and home/away site on survival **(A)** and biomass **(B)**. Home: plants in their home site, away: plants in away sites. Values represent the means ± 1 standard error (SE). Significance level *p* < 0.05 (^*^).

Survival and biomass of plants transplanted to their home environment significantly differed according to the country and temperature of origin (Country × Temperature.Origin × Home, Table 2A). For survival, we found evidence of local maladaptation for the warm populations in all countries (Figure 1A). In the warm sites, survival of plants was consistently lower for plants transplanted to their home than away sites. On the other hand, in the intermediate sites, survival was higher for the populations in their home environment both in Italy and Norway, while it did not differ in Czechia. Lastly, survival was higher for the home population in the cold sites in Italy and Czechia, but did not differ in Norway.

Biomass of surviving plants from the warm populations did not significantly differ among home and away plants (Figure 1B). Plants from the intermediate sites tended to have higher biomass when transplanted to their home than away sites in all three countries. Finally, plants from the cold sites tended to have lower biomass when transplanted to their home than away sites in all countries but significantly only in Italy.

### Is Local Adaptation Mediated by DNA Methylation? (Hypothesis 2)

**Figure 2:**
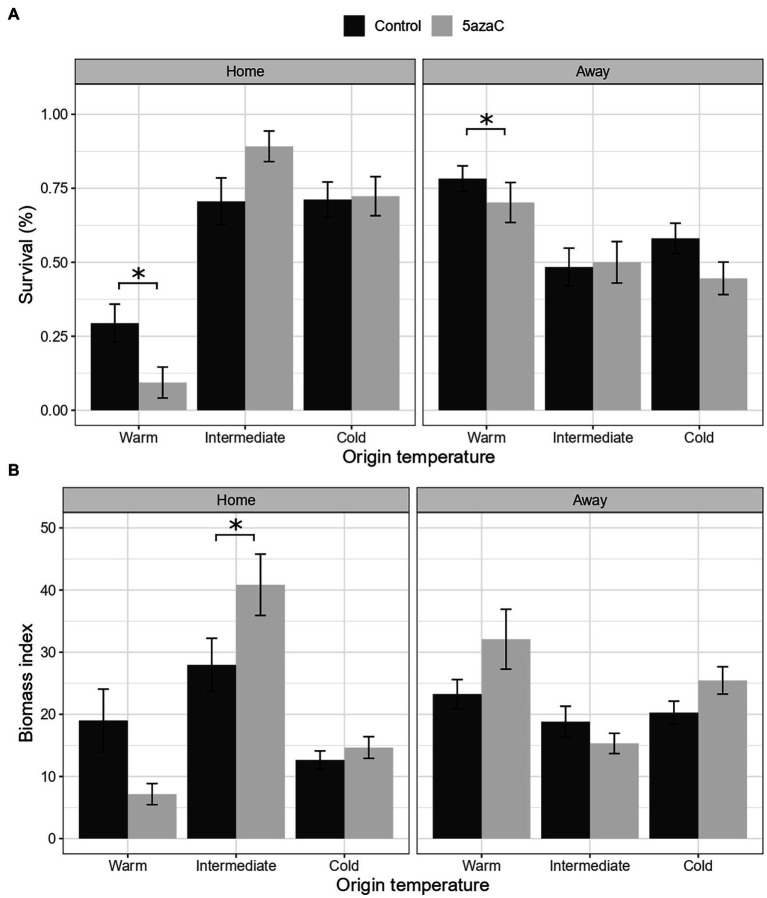
Is local adaptation mediated by DNA methylation (Hypothesis 2)? Effects of origin temperature, home/away site and 5-azaC on plant survival **(A)** and biomass **(B)**. Home: plants in their home site, away: plants in away sites. Control: plants with natural DNA methylation, 5azaC: plants treated with 5-azaC. Values represent the means ± 1 standard error (SE). Significance level *p* < 0.05 (^*^).

We found a significant interaction of 5-azaC with home-away effects for biomass and marginally significant for survival (Temperature.Origin × Home × 5-azaC, Table 2B). In the warm sites, both survival and biomass consistently decreased in 5-azaC-treated plants transplanted in their home sites compared to plants with natural DNA methylation (Figures 2A,B). In the other temperature sites, 5-azaC treatment had mostly no effect on plant survival and biomass, except for the populations from the intermediate sites transplanted in their home sites, in which biomass of 5-azaC-treated plants was higher than survival of plants with natural DNA methylation (Figures 2A,B).

For herbivory damage, we found significant effect of 5-azaC treatment alone and in combination with country (5-azaC; Country × 5-azaC, Table 2B), but no significant interaction of application of 5-azaC with home-away effects. Specifically, plants treated with 5-azaC showed increased levels of herbivory than control plants (mean ± SE, Ctrl = 0.36 ± 0.03, 5-azaC = 0.53 ± 0.04), and such a difference was weaker in Norway than in the other two countries (Supporting Information, Supplementary Figure S1).

### Is Adaptation to Warm Conditions Mediated Differently Than to Cold Conditions? (Hypothesis 3)

To test for the role of DNA methylation in response of transplantation to different climatic conditions in the three countries, we tested the effects of 5-azaC treatment in interaction with country and temperature distance between origin and target sites on plant survival, biomass and herbivory damage.

**Figure 3:**
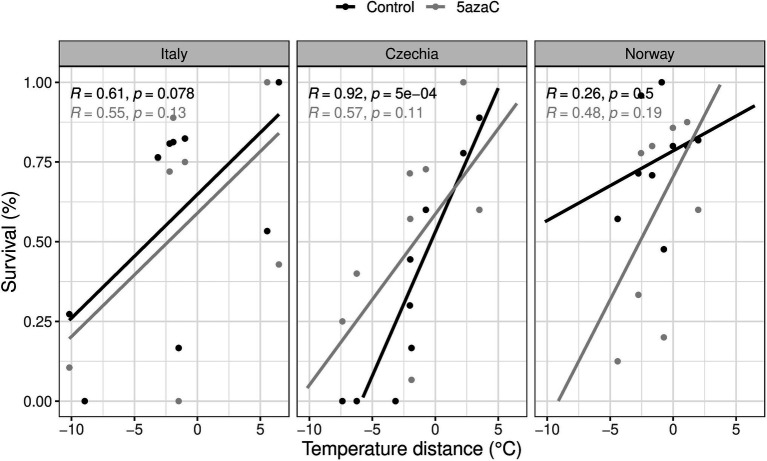
Is adaptation to warm and/or cold conditions mediated by DNA methylation (Hypothesis 3)? Effects of country, temperature distance and 5-azaC on plant survival. Positive values represent shift to colder sites, negative values to warmer sites. Control: plants with natural DNA methylation, 5azaC: plants treated with 5-azaC. R: Pearson correlation, *p*: *p*-value.

We found no significant interactions of 5-azaC treatment with temperature distance and/or country on either plant biomass or herbivory damage (Table 3). However, we found a significant effect on survival of 5-azaC treatment in interaction with both country and temperature distance (Country x Temperature.Distance x 5-azaC, Table 3). Specifically, in all the countries, survival consistently increased for plants transplanted from warmer to colder sites, while the effect of the 5-azaC treatment was country-specific (Figure 3). For both Italy and Czechia, the correlation between survival and temperature distance was stronger in plants with natural DNA methylation than 5-azaC plants, while in Norway it was stronger in 5-azaC than plants with natural DNA methylation.

**Table 3:** Test of role of DNA methylation in adaptation to warm and/or cold conditions (Hypothesis 3).

		Survival		Biomass		Herbivory damage	
	d.f.	F	*P*	F	*P*	F	*P*
Country (C)	2	0.94	0.364	1.01	0.411	0.25	0.808
Temperature.Distance (T)	1	0.79	0.206	0.17	0.692	2.25	0.237
5-azaC	1	2.45	0.102	0.16	0.693	**10.22**	**0.001**
C × T	2	**2.55**	**0.035**	0.32	0.740	1.70	0.211
C × 5-azaC	2	**2.96**	**0.048**	0.91	0.402	0.03	0.998
T × 5-azaC	1	0.01	0.979	0.04	0.837	0.84	0.357
C × T × 5-azaC	2	**4.43**	**0.008**	0.38	0.681	0.61	0.542

Effects of country of origin (Country, C), temperature distance (Temperature.Distance, T), 5-azaC treatment (5-azaC), and their interactions on plant survival, biomass, and herbivory damage. *N* = 730 (survival), *N* = 403 (biomass, herbivory damage). D.f.: degrees of freedom. Significant values (*p* ≤ 0.05) are shown in bold. Estimates of the effects can be found in (Supporting Information, Supplementary Table S4).

## Discussion

The ongoing climate crisis that is threatening plants and other organisms triggered an intense debate on whether and how plants will be able to adapt to future environmental conditions. By using a reciprocal transplant experiment, we tested whether *Fragaria vesca* is adapted to local conditions in three European countries across a climatic gradient and if it performs better or worse to increasing temperatures. We measured survival, biomass, and herbivory damage of transplanted ramets, and we consider survival as the main proxy of performance since this is the ultimate measure of plant fitness. Based on survival, we detected local adaptation only for the intermediate and cold populations in Italy. On the other hand, we found evidence for maladaptation of *F. vesca* to warm temperature in all countries (Figure 1A). Moreover, based on biomass, we found evidence for maladaptation for the cold population in Italy (Figure 1B). By experimental alteration of DNA methylation of selected plants, we also tested whether local adaptation can be under DNA methylation control. Ramets with altered DNA methylation (5-azaC, about 27% reduced overall DNA methylation in comparison to controls) showed worse performance (significant only for survival) in their local conditions than untreated plants, but only in the warm sites, suggesting that DNA methylation plays particularly a role in response to warmer temperatures (Figure 2). Finally, we tested whether DNA methylation plays a distinct role in adaptation to contrasting environments (warm/cold conditions). Both plants with natural DNA methylation and plants with altered DNA methylation showed a positive correlation between survival and temperature distance between the origin and target sites, but the effect of the 5-azaC treatment was country-specific (Figure 3).

### Local Adaptation and Response to Climate Change

Regarding the survival of transplanted ramets, our data revealed that all populations from the warm localities were maladapted to their home environment. On the other hand, we found no evidence of maladaptation to intermediate or cold temperature conditions in all countries, suggesting that the limiting factor for the survival of *F. vesca* were high summer temperatures. The maladaptation observed in the warm populations might be due to the exceptionally high summer temperatures that our populations experienced in the transplantation year, which might have crossed physiological limits enabling them to survive. Interestingly, the cold Italian population showed local adaptation if survival is considered but maladaptation in the case of biomass. We have no clear explanation for such contrasting response. It is possible that lower biomass could be ascribed to higher survival rate of smaller plants. However, more research would be needed to uncover the cause of this discrepancy.

It is also important to acknowledge that *F. vesca* usually forms large systems of interconnected ramets that can exchange resources as well as information, which was not possible to address in our study. In theory, plants in warm and/or dry localities might benefit from among-ramets resource sharing more than plants from cold and/or wet localities due to potential water reallocation. Individual ramets can be specialized for water acquisition due to division of labor, which can enhance performance of the whole clone (Alpert and Stuefer, 1997). Therefore, the observed ramet maladaptation in warm localities could be mitigated or even absent in situations when clones consist of more interconnected ramets. This possibility remains to be elucidated in future studies.

The lack of local adaptation found in Czechia and Norway may stem from several reasons. First, we could miss the evidence of local adaptation at other developmental stages, for example at the level of reproduction of transplanted plants as only very few plants flowered in the experiment. Local adaptation could be also better expressed over longer period, typically during overwintering, which was not a part of our study. According to Germino et al. (2019), proper testing of local adaptation requires a longer time to become evident, sometimes even decades. The absence of local adaptation could be attributed to the relatively low standing genetic variation of our populations, visible from low allelic richness ranging from 1.52 to 1.74 and observed heterozygosity lower than the expected under Hardy–Weinberg equilibrium, and same genotypic diversity among populations (Shannon–Wiener Index of MLG diversity; Sammarco et al., unpublished). Low genetic variability, inbreeding depression and genetic drift can in fact hinder local adaptation of small populations (<1,000 flowering individuals; Leimu and Fischer, 2008). Finally, the lacking evidence for local adaptation was found also in many other studies (e.g., Ebeling et al., 2011; Tíscar et al., 2018; Anderson and Wadgymar, 2020), as was evidence for maladaptation (reviewed in Brady et al., 2019), suggesting that local adaptation might be surprisingly rare to find in the wild.

In general, survival decreased in plants transplanted to warmer localities and increased when these were transplanted to colder localities. The negative effect of increased temperature on plant survival is perhaps a more general pattern as this phenomenon has been found for other plant species as well (e.g., Birami et al., 2018; Hammer et al., 2018). However, it is worth stressing that such a pattern does not seem to be universal. For example, in a meta-analysis of several mountain species including forbs, graminoids, and trees, the authors found no difference in survival for individuals transplanted at elevations lower than the site of origin, but lower survival for individuals transplanted at higher elevations (Midolo and Wellstein, 2020). In rare cases, plants can even thrive better in warmer sites than was their origin, and this can be ascribed to competitive release (Lenoir et al., 2010) or other climate-related factors than to the increased temperature *per se* (Dobrowski et al., 2013; Rapacciuolo et al., 2014; Putnam and Reich, 2017). Thus, plant response to climatic shifts may be species- or life-history-specific, and/or depend on the specific environmental conditions.

It is not only temperature but also the change in precipitations that can contribute to the success or failure of plants in climatically different environments (Midolo and Wellstein, 2020). Plant response to climatic shifts might also depend on the species’ distribution optima. In fact, colonization success has been shown to increase for species with warmer distribution optima than the target site, and to decrease for species with colder distribution optima (Reich et al., 2015; Liu et al., 2018; Lynn et al., 2021). We are not able to completely disentangle the temperature and precipitations effects on plant survival due to the correlation between the two factors. Nonetheless, in our study, the precipitation change did not explain survival of transplanted ramets better than the temperature suggesting that the primary driving factor for survival was the temperature change, rather than the precipitation change.

### Epigenetic Variation in Local Adaptation

In the populations from the warm sites, both survival and biomass significantly decreased in 5-azaC-treated plants when transplanted to their home environment (Figure 2). Better survival and biomass of plants that were not treated with 5-azaC in warm localities may be at least partly explained by contribution of DNA methylation to adaptation to warm conditions that was interfered by 5-azaC application. Considering that we did not observe such a pattern in other temperature conditions, we speculate that DNA methylation played different roles in adaptation to warmer and colder climatic conditions. In agreement with our findings, heat and cold stresses can affect plant epigenome differently. In many plant species, heat stress induces a global reduction in methylation level of DNA (i.e., hypomethylation; Ci et al., 2015; Li et al., 2016; Hossain et al., 2017), while cold stress causes a global increase in methylation level of DNA (i.e., hypermethylation; Pan et al., 2011; Ci et al., 2015). However, in species such as upland cotton (*Gossypium hirsutum*) or rubber trees (*Hevea brasiliensis*), cold treatment induces demethylation of genes involved in cold tolerance (Fan et al., 2013; Tang et al., 2018). Despite that the epigenetic response to heat and cold stresses seems to be species-specific, these results together with our study suggest that warm and cold climatic conditions shape plant epigenomes differently, meaning that the role of DNA methylation in response to different temperatures can vary.

To our knowledge, there is currently only one other study, Herden et al. (2019), investigating the effect of DNA methylation in local adaptation *via* experimental modification of plant methylome. The authors did not find evidence of local adaptation in several plant species but they also found no evidence of the role of DNA methylation in local adaptation. However, in the study of Herden et al. (2019), plants with experimentally altered methylomes were always smaller than control plants, suggesting negative side effects of methylation alteration during germination of the plants in the demethylating solution. Indeed, the significant reduction in plant growth has been already observed in other studies employing a similar approach for altering plant methylome (e.g., Ruiz-García et al., 2005; Akimoto et al., 2007; Kondo et al., 2007; Bossdorf et al., 2010). Thus, the lack of evidence of the role of DNA methylation in local adaptation found in Herden et al. (2019) might be due to the negative side effects associated with the demethylation approach, rather than by an actual lack of importance of DNA methylation in plant local adaptation. Instead, in our study, we can exclude the negative association between demethylation treatment and local adaptation, since we did not observe any negative side effects of application of 5-azaC on plant biomass. On the contrary, we have shown that the 5-azaC plants were even on average bigger than the plants with natural DNA methylation (Supplementary Note). This is in line with other studies using foliar application of 5-azaC for altering DNA methylation level of plants (e.g., González et al., 2016; Puy et al., 2018; Rendina González et al., 2018; Münzbergová et al., 2019). Our findings thus support the foliar application of 5-azaC over using it during seed germination, which provides a big advantage for plant ecological epigenetics studies (Puy et al., 2018). As the foliar application approach does not require growing plants from seeds, it allows alteration of DNA methylation level even on fully developed plants, thus enabling to work for example with a genetically uniform background in case of clonal plants.

### Epigenetics in Response to Climate Change

Climatic changes affect epigenetic variation in many organisms, which might help them adapt to rapid climatic changes (e.g., Gugger et al., 2016; Chano et al., 2021; reviewed in Thiebaut et al., 2019). In our study, however, experimental alteration of DNA methylation had inconsistent effects on the plant response to climate change in the three countries. While 5-azaC had virtually no effect on plant survival in response to temperature change in Italy, 5-azaC had contrasting effect on climate change in Czechia and Norway. Compared to plants with natural DNA methylation, application of 5-azaC reduced plant survival if plants were moved to warmer conditions in Norway but increased survival of plants moved to warmer conditions in Czechia. This might imply that removal of epigenetic memory on the original environment changed plant’s ability to survive their shift to climatically different localities. The contrasting effect of 5-azaC among Czech and Norwegian populations and the lack of effect on Italian populations on survival in changing climate suggests that the effect of DNA methylation is dependent on the local environmental conditions and/or on specific characteristics of the populations. For example, clones consisting of fewer ramets might have less division of labor, with each ramet expressing more generalist methylomes able to better respond to transplantations. This could be accompanied by variable effects of 5-azaC on plant’s methylome. We know that the methylome of treated plants was highly variable across localities (Sammarco et al., unpublished), suggesting that 5-azaC might have different effects in different localities. Nevertheless, the speculation needs to be tested in further studies. Alternatively, the different effect of 5-azaC might be due to genetic differences of plant populations between localities and, even more, between regions (5-azaC effect can be genotype-specific, Münzbergová et al., 2019). Furthermore, demethylation is also random and can be accompanied by activation of epigenetically silenced genes (Feng et al., 2010) or transposons (Griffin et al., 2016; Boonjing et al., 2020), which can together and in an unpredictable way affect behavior and thus also survival of 5-azaC-treated plants, which might be at least partly responsible for the different effect of 5-azaC in different regions of Europe.

### Future Outlooks

In order to provide unambiguous and strong data for generalization, we need studies similar to this one encompassing more species and populations over longer time periods. The studies should be also accompanied by sophisticated molecular methods such as whole-genome bisulfite sequencing. These can provide insights into the epigenetic variation of the plants. Experimental demethylation is however still crucial in such studies as molecular methods provide only indirect evidence of the role of epigenetic variation in local adaptation. Thus, even if the alteration of DNA methylation by 5-azaC occurs randomly and varies among individuals, it is still an important practical approach to provide direct evidence of the role of epigenetic variation in local adaptation when coupled with reciprocal transplant experiments. In fact, random demethylation of 5-azaC reduces the likelihood of identifying significant effects of 5-azaC treatment. Despite this potential for demethylation noise, our study observed significant effects of 5-azaC, suggesting a strong regulatory role played by DNA methylation. Finally, it is also likely that the role of epigenetic variation plays different roles at the level of individual ramet and whole clone. It is known that epigenetic variation can be greatly variable even within individual non-clonal plants (e.g., Herrera and Bazaga, 2013; Herrera et al., 2021). Considering clonal plants, it was proposed that differences in DNA methylation among communicating ramets could enhance whole genet functioning (Latzel et al., 2016), suggesting that the observed responses of single ramets may not be scalable to whole plant generalizations. Therefore, future studies should try incorporate larger parts of individual clones.

## Conclusion

Our study is among the first testing the role of DNA methylation in local adaptation by employing experimental demethylation in natural conditions across a broad scale of local climatic conditions. It provides evidence that epigenetic variation may contribute to adaptation to local conditions in natural ecosystems. Results of our study also suggest that the increasing temperature will be highly probably the limiting factor determining *F. vesca* survival, which can alter the distribution of the species in case of further temperature increases. By experimental alteration of DNA methylation, we also provided one of the first evidence that epigenetic variation can alter plant response to changing climatic conditions. Since adaptation mediated by epigenetic variation may occur faster than *via* random genetic processes, epigenetic adaptation might provide clonal plants with the necessary time to tackle ongoing environmental crisis and genetically adapt to it afterwards.

## Data Availability Statement

The raw data supporting the conclusions of this article will be made available by the authors, without undue reservation.

## Author Contributions

All authors listed have made a substantial, direct, and intellectual contribution to the work and approved it for publication.

## Funding

The study was supported by the European Union’s Horizon 2020 research and innovation programme under the Marie Skłodowska-Curie grant agreement No 764965, the Czech Science Foundation (GACR 20-00871S) and partly by institutional research project RVO 67985939.

## Conflict of Interest

The authors declare that the research was conducted in the absence of any commercial or financial relationships that could be construed as a potential conflict of interest.

## Publisher’s Note

All claims expressed in this article are solely those of the authors and do not necessarily represent those of their affiliated organizations, or those of the publisher, the editors and the reviewers. Any product that may be evaluated in this article, or claim that may be made by its manufacturer, is not guaranteed or endorsed by the publisher.
