# Supplementary material for: DNA Methylation Can Mediate Local Adaptation and Response to Climate Change in the Clonal Plant Fragaria vesca: Evidence From a European-Scale Reciprocal Transplant Experiment
Source: Front Plant Sci. 2022 Feb 28;13:827166. doi: 10.3389/fpls.2022.827166 (PMC8919072; doi:10.3389/fpls.2022.827166)

DNA methylation can mediate local adaptation and response to climate change in the clonal plant *Fragaria vesca*: evidence from a European-scale reciprocal transplant experiment

**Supplementary material**

Supplementary Note

*Effect of 5-azaC treatment on adaptation to precipitation change (Hypothesis 3)*

We tested the effects of 5-azaC treatment in interaction with country and precipitation distance between origin and target sites on plant survival, biomass and herbivory damage.

As for the test including temperature distance, we found no significant interactions of 5-azaC treatment with precipitation distance and/or country on either plant biomass or herbivory damage, but we found a significant effect on survival of 5-azaC treatment in interaction with both country and precipitation distance (Country x Precipitation.Distance x 5-azaC, F=2.94, p=0.049).

*Control for transplantation and 5-azaC effects*

In order to control for potential effects of the transplantation procedure and precultivation of plants in the common garden, together with the transplanted plants, we always re-planted between 8-10 plants found at each of the transplantation site (replanted local plants) at all localities but the cold locality in Czechia. Overall, from the comparison of survival and biomass of replanted local plants and precultivated plants not treated by 5-azaC (*i.e.* control plants), the transplantation had no considerable negative effects on plants survival and biomass (survival p=0.81, biomass p=0.78). We only observed a consistent difference in plant survival in all warm localities, with the survival of replanted local plants higher than the control plants. Such a finding might be explained by the exceptional warm summer the plants were exposed to in the transplantation year, which did not allow the transplanted plants to quickly adapt when moved from the favourable environment of the common garden to such extreme climatic conditions. The climatic conditions were indeed more extreme in the warm localities, and the difference in survival between the replanted local plants and the control plants was more evident in the hottest of the warm sites (Czech warm site), and less in the mildest of the warm sites (Norwegian warm site).

We also assessed whether the 5-azaC treatment induced possible negative side-effects on plant survival and/or biomass. We compared survival and biomass of transplanted plants with natural DNA methylation with those treated with 5-azaC. 5-azaC had no effect on plant survival (mean ± SE, Ctrl = 0.61 ± 0.02, 5-azaC = 0.56 ± 0.03; χ^2^= 0.22) but had a positive effect on plant biomass (mean ± SE, Ctrl = 20.34 ± 1.09, 5-azaC = 25.71 ± 1.70). In our study, we can therefore exclude that the 5-azaC treatment had negative side-effects on plant survival or biomass.

Supporting information

**Table S1: Experimental design of the reciprocal transplantations.** We collected 5 to 7 ramets from nine populations distributed along a climatic gradient from Italy, Czechia, Norway (“Origin”), and we transplanted them in their home locality (*) and in the other two away sites within the country of their origin (“Target”). The transplanted plants included both control plants and plants treated with 5-azaC (“Ramets”). Numbers in bracket indicate number of plants used in this study, *i.e.* number of maternal plants collected for each population (“Origin”) and number of clonal plants originated from those (“Ramets”) and transplanted in each target locality.

| **ITALY** | | | **CZECHIA** | | | **NORWAY**  **RAMETS** | | |
| --- | --- | --- | --- | --- | --- | --- | --- | --- |
| **Origin** | **Target** | **Ramets ramets** | **Origin** | **Target** | **Ramets ramets** | **Origin** | **Target** | **Ramets ramets** |
| Warm  (6) | Warm* | Ctrl (18) | Warm  (6) | Warm* | Ctrl (12) | Warm  (6) | Warm* | Ctrl (25) |
|  |  | 5-azaC (7) |  |  | 5-azaC (15) |  |  | 5-azaC (12) |
|  | Intermediate | Ctrl (16) |  | Intermediate | Ctrl (10) |  | Intermediate | Ctrl (21) |
|  |  | 5-azaC (6) |  |  | 5-azaC (11) |  |  | 5-azaC (8) |
|  | Cold | Ctrl (16) |  | Cold | Ctrl (9) |  | Cold | Ctrl (22) |
|  |  | 5-azaC (7) |  |  | 5-azaC (11) |  |  | 5-azaC (8) |
| Intermediate (7) | Warm | Ctrl (16) | Intermediate (5) | Warm | Ctrl (8) | Intermediate (5) | Warm | Ctrl (7) |
|  |  | 5-azaC (10) |  |  | 5-azaC (5) |  |  | 5-azaC (9) |
|  | Intermediate* | Ctrl (18) |  | Intermediate* | Ctrl (10) |  | Intermediate* | Ctrl (9) |
|  |  | 5-azaC (18) |  |  | 5-azaC (7) |  |  | 5-azaC (12) |
|  | Cold | Ctrl (17) |  | Cold | Ctrl (9) |  | Cold | Ctrl (6) |
|  |  | 5-azaC (16) |  |  | 5-azaC (4) |  |  | 5-azaC (10) |
| Cold  (7) | Warm | Ctrl (22) | Cold  (6) | Warm | Ctrl (4) | Cold  (6) | Warm | Ctrl (26) |
|  |  | 5-azaC (19) |  |  | 5-azaC (4) |  |  | 5-azaC (18) |
|  | Intermediate | Ctrl (26) |  | Intermediate | Ctrl (5) |  | Intermediate | Ctrl (18) |
|  |  | 5-azaC (25) |  |  | 5-azaC (5) |  |  | 5-azaC (19) |
|  | Cold* | Ctrl (21) |  | Cold* | Ctrl (9) |  | Cold* | Ctrl (28) |
|  |  | 5-azaC (22) |  |  | 5-azaC (7) |  |  | 5-azaC (17) |

**Table S2: Correlation between 5-azaC effect and genetic relationship between plants**. We found no significant correlation (*P* ≤0.05) between 5-azaC effect and genetic relatedness, suggesting that the effect of 5-azaC did not differ between genotypes. Correlation (r) was measured using the Spearman method for the full Mantel tests (no covariate), and Pearson method for the partial Mantel tests (covariates).

|  | Survival (n = 98) | | Biomass (n = 59) | |
| --- | --- | --- | --- | --- |
| Covariate | Correlation (r) | *P* | Correlation (r) | *P* |
| None | -0.02 | 0.68 | 0.02 | 0.70 |
| Distance of origin temperatures | -0.02 | 0.95 | -0.01 | 0.73 |
| Distance of target temperatures | -0.02 | 0.95 | -0.02 | 0.90 |
| Same origin locality | 0.01 | 0.18 | -0.05 | 0.99 |
| Same target locality | -0.02 | 0.96 | -0.02 | 0.91 |

**Table S3: Estimates of the effects of country (Country, Czechia used as baseline), temperature of origin (Temperature.Origin, intermediate used as baseline), home/away site (Home), 5-azaC treatment (5-azaC) and their interactions on plant survival, biomass and herbivory damage.**

|  | Survival | Biomass | Herbivory damage |
| --- | --- | --- | --- |
| Country (Italy) | 0.05 | -0.87 | -0.29 |
| Country (Norway) | 1.46 | -0.65 | 0.41 |
| Temperature.Origin (warm) | 1.39 | -0.27 | -0.88 |
| Temperature.Origin (cold) | -18.06 | 1.31 | 2.00 |
| Home | -0.49 | 0.24 | -0.98 |
| 5-azaC | 0.36 | -0.87 | -1.67 |
| Country (Italy) x Temperature.Origin (warm) | 0.15 | 0.65 | 0.18 |
| Country (Norway) x Temperature.Origin (warm) | -1.04 | 0.44 | -0.05 |
| Country (Italy) x Temperature.Origin (cold) | 18.31 | -0.66 | -2.11 |
| Country (Norway) x Temperature.Origin (cold) | 18.21 | -1.33 | -3.76 |
| Country (Italy) x Home | 2.26 | 0.36 | 0.51 |
| Country (Norway) x Home | 17.82 | 0.19 | -0.81 |
| Temperature.Origin (warm) x Home | -2.15 | 0.25 | -14.97 |
| Temperature.Origin (cold) x Home | 18.68 | -2.04 | -2.41 |
| Country (Italy) x 5-azaC | -0.21 | 1.08 | 4.07 |
| Country (Norway) x 5-azaC | -1.20 | 0.84 | 1.20 |
| Temperature.Origin (warm) x 5-azaC | -0.69 | 1.15 | 2.85 |
| Temperature.Origin (cold) x 5-azaC | 17.37 | -0.50 | 0.08 |
| Home x 5-azaC | 1.41 | 1.56 | 2.77 |
| Country (Italy) x Temperature.Origin (warm) x Home | -2.46 | -0.35 | 16.83 |
| Country (Norway) x Temperature.Origin (warm) x Home | -16.72 | -1.47 | 15.61 |
| Country (Italy) x Temperature.Origin (cold) x Home | -18.97 | 0.55 | 1.54 |
| Country (Norway) x Temperature.Origin (cold) x Home | -36.37 | 0.98 | 4.65 |
| Country (Italy) x Temperature.Origin (warm) x 5-azaC | 0.12 | -1.32 | -5.81 |
| Country (Norway) x Temperature.Origin (warm) x 5-azaC | 1.30 | -1.00 | -1.74 |
| Country (Italy) x Temperature.Origin (cold) x 5-azaC | -17.67 | 0.47 | -2.59 |
| Country (Norway) x Temperature.Origin (cold) x 5-azaC | -17.89 | 0.66 | 0.94 |
| Country (Italy) x Home x 5-azaC | -0.95 | -1.61 | -4.45 |
| Country (Norway) x Home x 5-azaC | -0.52 | -1.03 | -1.54 |
| Temperature.Origin (warm) x Home x 5-azaC | -2.10 | -3.53 | -8.94 |
| Temperature.Origin (cold) x Home x 5-azaC | -18.62 | -0.24 | -0.08 |
| Country (Italy) x Temperature.Origin (warm) x Home x 5-azaC | -18.82 | / | / |
| Country (Norway) x Temperature.Origin (warm) x Home x 5-azaC | 0.17 | 2.49 | -15.14 |
| Country (Italy) x Temperature.Origin (cold) x Home x 5-azaC | 17.82 | 0.15 | 3.54 |
| Country (Norway) x Temperature.Origin (cold) x Home x 5-azaC | 19.60 | / | / |

**Table S4: Estimates of the effects of country (Country, Czechia used as baseline), temperature distance (Temperature.Distance), 5-azaC treatment (5-azaC) and their interactions on plant survival, biomass and herbivory damage.**

|  | Survival | Biomass | Herbivory damage |
| --- | --- | --- | --- |
| Country (Italy) | 0.488 | -0.334 | 0.159 |
| Country (Norway) | 1.341 | -0.655 | 0.123 |
| Temperature.Distance | 0.535 | -0.026 | 0.084 |
| 5-azaC | 0.090 | -0.130 | 0.664 |
| Country (Italy) x Temperature.Distance | -0.550 | 0.040 | -0.156 |
| Country (Norway) x Temperature.Distance | -0.661 | 0.081 | 0.025 |
| Country (Italy) x 5-azaC | -0.419 | 0.197 | -0.035 |
| Country (Norway) x 5-azaC | -0.341 | 0.288 | 0.180 |
| Temperature.Distance x 5-azaC | -0.407 | 0.026 | -0.191 |
| Country (Italy) x Temperature.Distance x 5-azaC | 0.424 | -0.051 | 0.093 |
| Country (Norway) x Temperature.Distance x 5-azaC | 0.742 | -0.007 | 0.313 |

**Figure S1: Effects of country and 5-azaC on herbivory damage.** Control: plants with natural DNA methylation, 5azaC: plants treated with 5-azaC. Values represent the means ± 1 standard error (SE). Significance level p < 0.05.


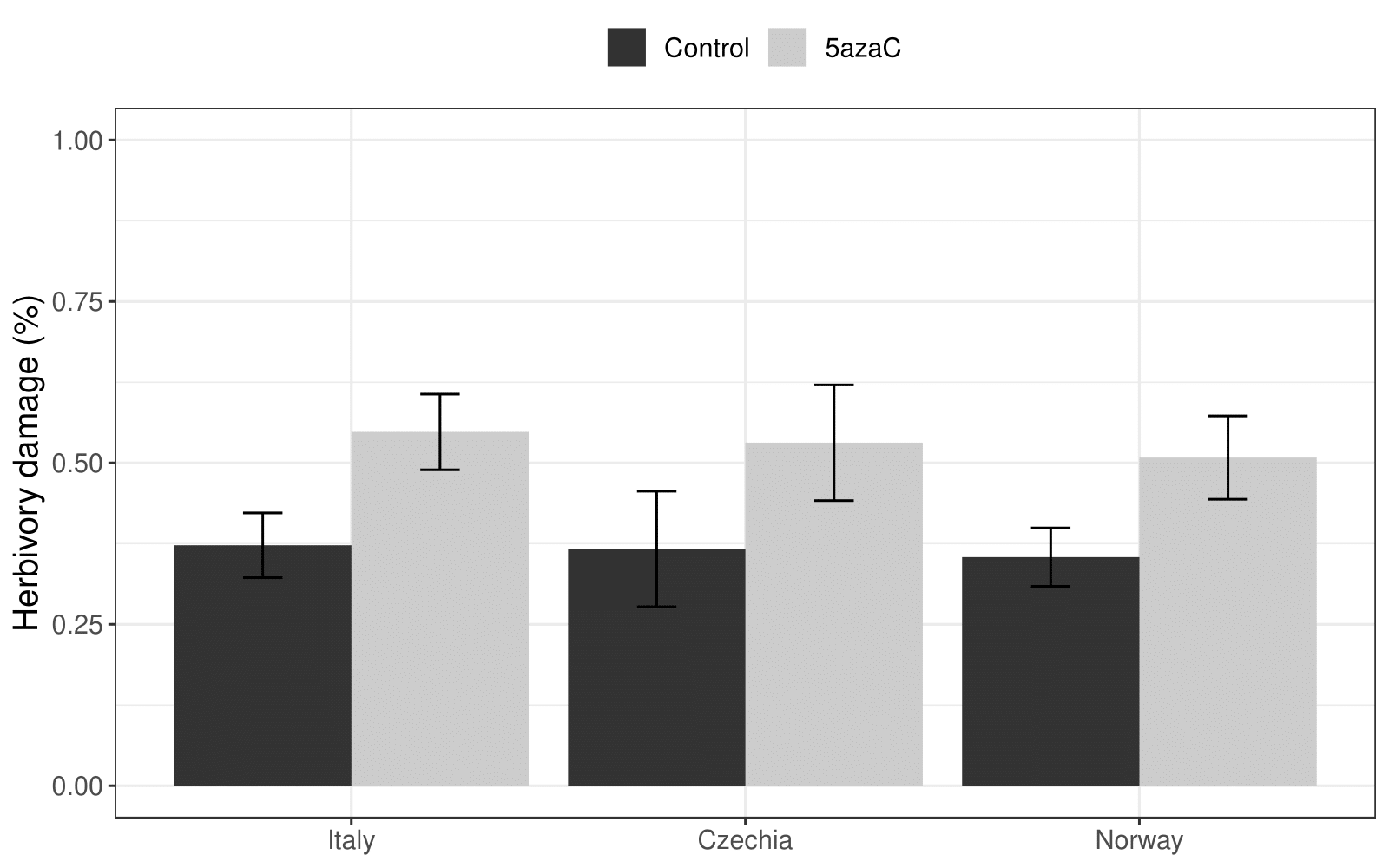

Supplement: Supplementary file 1 [file Data_Sheet_1.docx]
